# Ubiquitination-Dependent Regulation of Small GTPases in Membrane Trafficking: From Cell Biology to Human Diseases

**DOI:** 10.3389/fcell.2021.688352

**Published:** 2021-07-01

**Authors:** Zehui Lei, Jing Wang, Lingqiang Zhang, Cui Hua Liu

**Affiliations:** ^1^CAS Key Laboratory of Pathogenic Microbiology and Immunology, Institute of Microbiology, Center for Biosafety Mega-Science, Chinese Academy of Sciences, Beijing, China; ^2^Savaid Medical School, University of Chinese Academy of Sciences, Beijing, China; ^3^State Key Laboratory of Proteomics, Beijing Proteome Research Center, National Center for Protein Sciences (Beijing), Beijing Institute of Lifeomics, Beijing, China

**Keywords:** small GTPase, ubiquitination, membrane trafficking, neurological diseases, infections

## Abstract

Membrane trafficking is critical for cellular homeostasis, which is mainly carried out by small GTPases, a class of proteins functioning in vesicle budding, transport, tethering and fusion processes. The accurate and organized membrane trafficking relies on the proper regulation of small GTPases, which involves the conversion between GTP- and GDP-bound small GTPases mediated by guanine nucleotide exchange factors (GEFs) and GTPase-activating proteins (GAPs). Emerging evidence indicates that post-translational modifications (PTMs) of small GTPases, especially ubiquitination, play an important role in the spatio-temporal regulation of small GTPases, and the dysregulation of small GTPase ubiquitination can result in multiple human diseases. In this review, we introduce small GTPases-mediated membrane trafficking pathways and the biological processes of ubiquitination-dependent regulation of small GTPases, including the regulation of small GTPase stability, activity and localization. We then discuss the dysregulation of small GTPase ubiquitination and the associated human membrane trafficking-related diseases, focusing on the neurological diseases and infections. An in-depth understanding of the molecular mechanisms by which ubiquitination regulates small GTPases can provide novel insights into the membrane trafficking process, which knowledge is valuable for the development of more effective and specific therapeutics for membrane trafficking-related human diseases.

## Introduction

Membrane trafficking along with the endocytic, exocytic and autophagic pathways ensures the flow of membranes and cargoes (which contain proteins, nutrients or other molecules) between different compartments within cells, and thus plays a critical role in cellular homeostasis. These complex membrane trafficking events are mostly regulated by small GTPases, which are divided into five families: Ras sarcoma (Ras), Ras homologous (Rho), Ras-like proteins in brain (Rab), ADP-ribosylation factor (Arf), and Ras-like nuclear (Ran) proteins (Wennerberg et al., 2005). The Rab family comprises approximately 60 members that are localized on distinct membranes (Wandinger-Ness and Zerial, 2014), and these proteins are the master modulators of membrane trafficking pathways (Pfeffer, 2017). The Arf and Arf-like (Arl) families also play a critical role in membrane trafficking along with the endocytic and exocytic pathways (Gillingham and Munro, 2007; Donaldson and Jackson, 2011; Yu and Lee, 2017; Kjos et al., 2018). Moreover, recent studies have demonstrated that the Rho as well as Ras families are also involved in membrane trafficking-related processes. For instance, Rho GTPases are required for the endocytic and exocytic pathways (Olayioye et al., 2019), while Ras GTPases mainly function in exocytic and autophagic pathways (Simicek et al., 2013; Nishida-Fukuda, 2019).

The basis of small GTPases to exert their functions is the conversion between GDP- and GTP-bound forms catalyzed by guanine nucleotide exchange factors (GEFs) and GTPase-activating proteins (GAPs) (Stenmark, 2009). Generally, GTP-bound form is considered to be the active state of GTPase, which can recruit specific effectors to regulate cellular activities, while GDP-bound form is the inactive state of GTPase that is usually restricted in the cytosol till being activated. Furthermore, increasing studies have shed light on the role of post-translational modifications (PTMs), mainly including phosphorylation, ubiquitination and prenylation, in the regulation of small GTPases (Ahearn et al., 2011; Hodge and Ridley, 2016; Shinde and Maddika, 2018). Among these modifications, ubiquitination is a highly conserved multistep enzymatic process catalyzed by ubiquitin-activating enzymes (E1s), ubiquitin-conjugating enzymes (E2s), and ubiquitin-ligase enzymes (E3s) sequentially, ultimately resulting in the attachment of single ubiquitin or multiple ubiquitin chains to target proteins. Ubiquitination is a critical signal to determine the stability, activity and localization of substrates, and thus is essential for regulating physiological functions of the substrates (Foot et al., 2017). Consistently, ubiquitination is critical for the spatio-temporal regulation of small GTPases, and this ubiquitination-dependent modulation is correlated with multiple human diseases (Qiu et al., 2016; Escamilla et al., 2017).

Here, we first provide an overview of small GTPases involved in membrane trafficking pathways. Then, we introduce the current knowledge on ubiquitination-dependent regulation of small GTPases. We also discuss human diseases associated with the dysregulation of small GTPase ubiquitination with a focus on neurological and infectious diseases. A better understanding of the ubiquitination-mediated regulation of small GTPases and its specific effects on membrane trafficking-related diseases will provide new insights into the therapeutics for these diseases.

## Small GTPases in Membrane Trafficking Pathways

**FIGURE 1:**
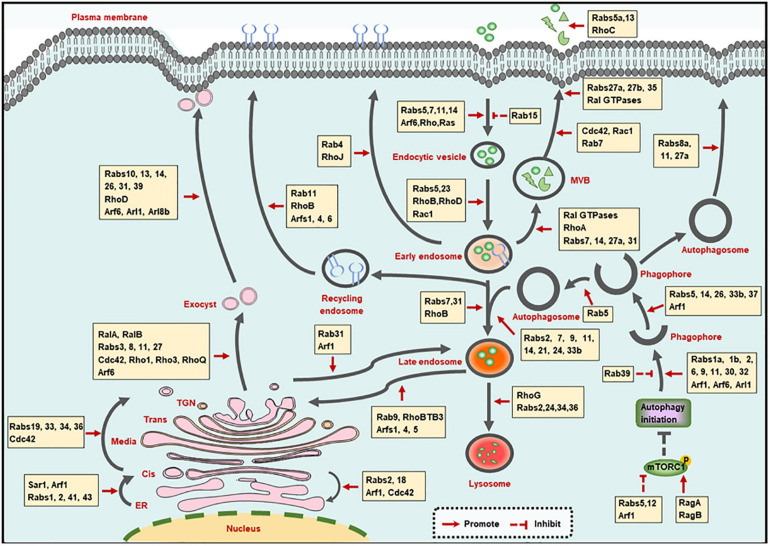
Small GTPases in membrane trafficking pathways. The illustration shows the main intracellular membrane trafficking pathways including endocytic, exocytic, and autophagic pathways regulated by small GTPases. TGN: trans-Golgi network; ER, endoplasmic reticulum; MVB, multivesicular bodies.

Membrane trafficking-mediated protein and membrane transport ensures the proceeding of endocytic, exocytic and autophagic pathways. Small GTPases, including Rab, Arf, Rho, and Ras families, play crucial roles in the membrane trafficking along with these pathways (Figure 1).

### Small GTPases in Endocytic Pathway

Small GTPases control the orderly proceeding of the trafficking steps involved in the endocytic pathway, which involves the following four steps: formation of endocytic vesicles via membrane internalization, trafficking of endocytic vesicles to early endosomes, trafficking of early endosomes to lysosomes, as well as trafficking of endosomes to the recycling compartments (Elkin et al., 2016).

### Membrane Internalization Step

Small GTPases are involved in the three modes of membrane internalization, including phagocytosis, pinocytosis and receptor-mediated endocytosis. Phagocytosis and pinocytosis are actin-dependent endocytic pathways and are mainly mediated by Rho as well as Rab GTPases, which have been summarized in elegant reviews (Hall, 2012; Egami, 2016). In addition, Ras can promote the translocation of vacuolar ATPase (V-ATPase) from intracellular membranes to the plasma membrane, followed by the stimulation of membrane ruffling and the occurrence of pinocytosis (Ramirez et al., 2019).

Clathrin-mediated endocytosis (CME) and clatherin-independent endocytosis (CIE) are routes of receptor-mediated endocytosis, and are mainly modulated by Rab and Arf GTPases, respectively. Specifically, Rab5 promotes the CME of transferrin receptor (TfR) (Stenmark et al., 1994), while Rab15 reduces the rate of TfR internalization, which may be caused by the decreased vesicle budding from the plasma membrane (Zuk and Elferink, 1999). Rab14 mediates the CME of the urea transporter A1 (UT-A1) (Su et al., 2013), and Rab5, Rab7, and Rab11 are required for virus intake through CME pathway (Shi et al., 2016; Liu et al., 2017). Additionally, the small GTPase Arf6, which is located at the cell surface in an active state to promote phospholipid metabolism, is the main modulator of CIE process (D’Souza-Schorey and Chavrier, 2006). Furthermore, Rab35 is involved in the communication and coordination between CME and CIE. The inhibition of CME shifts the sorting of CIE cargo proteins to lysosomes for degradation, and Rab35 can rescue the altered trafficking by recruiting the Arf6 GAP protein ACAP2 [Arf GAP, with Coil, ankyrin (ANK) repeat, pleckstrin homology (PH) domain protein 2] to inactivate Arf6 (Kobayashi and Fukuda, 2012; Dutta and Donaldson, 2015).

### Trafficking of Endocytic Vesicles to Early Endosomes

Upon internalization, the endocytic vesicles are transported to fuse with early endosomes, and are then sorted to decide their final fates (Huotari and Helenius, 2011). Since Rab5 and Rab23 are localized on both plasma membrane and early endosomes, they can mediate the trafficking of components between these two compartments by recruiting numerous effector proteins (Evans et al., 2003; Wandinger-Ness and Zerial, 2014). Besides Rab GTPases, Rho GTPases (such as RhoB, RhoD, and Rac1) that are located on the early endosomes, are also reported to be involved in cargoes transport to early endosomes (Olayioye et al., 2019). The identification of Rho effectors at the specific sites will extend our knowledge regarding the function of Rho GTPases in trafficking of endocytic vesicles to early endosomes.

### Trafficking of Early Endosomes to Lysosomes

The trafficking of early endosomes to lysosomes is tightly regulated by the transition of Rab5-to-Rab7 and Rab7-to-Arl8b. For the transition of early to late endosomes, Rab5 can recruit the Mon1/Ccz1 protein complex to promote Rab7 activation. Meanwhile, Rab5 GEF was removed by the Mon1/Ccz1 complex, resulting in the transition of Rab5-positive early endosome to Rab7-positive late endosomes (Poteryaev et al., 2010; Langemeyer et al., 2020). Consecutively, Rab7 is inactivated by Arl8b effector SKIP and is then removed from hybrid Rab7/Arl8b endosomes, leading to an ordered Rab7-to-Arl8b handover, followed by the fusion of late endosomes with lysosomes (Jongsma et al., 2020). Moreover, some other GTPases also participate in the early endosome to lysosomal transport. Rab31 and RhoB are required for the transition of early to late endosomes (Huang et al., 2007; Chua and Tang, 2014). Rab2 (Lund et al., 2018), Rab24 (Amaya et al., 2016), Rab34 (Wang and Hong, 2002), Rab36 (Chen et al., 2010), and RhoG (Vignal et al., 2001) are involved in the fusion of late endosomes with lysosomes.

Additionally, during the maturation process from early to late endosomes, multivesicular bodies (MVBs) are generated and then fused with plasma membrane for secretion, leading to the formation of exosomes. Various of small GTPases have been implicated in exosome biogenesis and secretion. Specifically, Ras-related (Ral) proteins (Hyenne et al., 2015), Rab7, Rab27a (Dorayappan et al., 2018), and RhoA (Li et al., 2012) contribute to the formation of MVBs; Rab31 drives MVB formation but suppresses their degradation simultaneously (Wei et al., 2021); Rab14 positively modulates the MVB diameter and number (Maziveyi et al., 2019). Additionally, Cdc42 and Rac1 promote MVB maturation (Kajimoto et al., 2018); and Rab7 mediates the transportation of MVBs (Sun et al., 2020). Moreover, Rab27a, 27b (Ostrowski et al., 2010), Rab35 (Hsu et al., 2010), and Ral GTPases (Pathak and Dermardirossian, 2013) function in the attachment of MVBs to the plasma membrane. Finally, Rab5a (Gorji-Bahri et al., 2021), Rab13 (Hinger et al., 2020), and RhoC (Hu et al., 2020) promote the secretion of exosomes.

### Trafficking of Endosomes to the Recycling Compartments

Endocytic vesicles from early endosomes and late endosomes can also be recycled to the plasma membrane and the trans-Golgi network (TGN), respectively. Basically, endocytic vesicles sorting from early endosomes can be recycled back to the plasma membrane by a fast or a slow route. Rab4 and Rab11 are the major regulators of the fast- and slow- recycling pathways, respectively (Wandinger-Ness and Zerial, 2014). Moreover, RhoB and RhoJ in Rho family GTPases, as well as Arf1, Arf4, and Arf6 in Arf family GTPases, are also involved in the recycling pathway from endosomes to the plasma membrane (de Toledo et al., 2003; Huang et al., 2011; Kondo et al., 2012; Grossmann et al., 2019). Significantly, the recycling pathways mediated by multiple GTPases are often crossing with each other. For instance, the Rab-to-Arf and Arf-to-Rab regulatory cascades have been reported. On the one hand, Rab10 recruits its effector CNT-1 (homolog of ACAP1/2 in *Caenorhabditis elegans*), which is also the GAP of Arf6, to suppress Arf6 activity, leading to the inhibition of membrane bending and membrane fission (Shi et al., 2012). On the other hand, the activated Arf6 can interact with the Rab35 GAP TBC1D10 to decrease Rab35 activity (Chesneau et al., 2012). These cascades ensure the ordered proceeding of complicated trafficking pathways.

Besides recycling to the plasma membrane, vesicles can also be sent to the TGN via the retrograde trafficking pathway from late endosomes. Rab9 that is located on the TGN and late endosomes mediates the recycling of mannose-6-phosphate receptors (MPRs) from late endosomes to TGN (Riederer et al., 1994; Kucera et al., 2016). And through interacting with Rab9, the atypical Rho GTPase family member RhoBTB3 is also involved in the retrograde trafficking pathway (Espinosa et al., 2009). Additionally, Arf, and Arl proteins, encompassing Arf1, Arf4, and Arl5, also contribute to endosome-to-TGN trafficking by recruiting different effectors (Nakai et al., 2013; Rosa-Ferreira et al., 2015).

### Small GTPases in Exocytic Pathway

The exocytic pathway involves the transporting between a series of membrane bound organelles, mainly including the transport from endoplasmic reticulum (ER) to Golgi, the transport within Golgi, the transport from Golgi to the cell surface, as well as transport from Golgi to the endocytic compartment. This dynamic and organized process transfers the synthesized proteins or other molecules into the cell surface or lysosomes in a small GTPase-dependent manner (Beraud-Dufour and Balch, 2002; Wu and Guo, 2015).

### ER-to-Golgi Transport

Upon being synthesized at the ER, proteins or other molecules need to exit from the ER and are then transferred to ER-Golgi intermediate compartment (ERGIC) and Golgi for further selection and transportation. The coat protein complex II (COPII) vesicles contribute to the ER exit, and small GTPase secretion-associated Ras-related 1 (Sar1) is required for the assembly of COPII vesicles (Nakano and Muramatsu, 1989). The assembled vesicles are then transported to ERGIC or Golgi. During this process, Rab1 cooperates with the tethering factors, and soluble NSF attachment protein receptor (SNARE) to promote the COPII vesicles transport to and fuse with Golgi (Allan et al., 2000; Moyer et al., 2001). Rabs2, 41, and 43 are also needed for rapid ER-to-Golgi trafficking (Dejgaard et al., 2008; Brandizzi and Barlowe, 2013; Liu et al., 2013; Li et al., 2017). Moreover, Arf1, which is primarily localized to the Golgi complex, is involved in the translocation of stimulator of interferon genes (STING) and soluble VEGFR-1 from ER to ERGIC or Golgi (Jung et al., 2012; Gui et al., 2019).

Once COPII vesicles are transported to Golgi, ER receptors and other ER proteins carrying a Lys-Asp-Glu-Leu (KDEL) motif are returned back to the ER through the COPI-mediated retrograde transport. This process is largely regulated by Arf1, since the recruitment of COPI is relied on the activated Arf1 (Serafini et al., 1991). Rab2, Rab18, and Cdc42 are also the modulators of the trafficking from Golgi to ER (Luna et al., 2002; Dejgaard et al., 2008; Brandizzi and Barlowe, 2013). It is worth mentioning that Rab2 and Arf1 mediate the bi-directional ER-Golgi trafficking, which may be achieved by recruiting different effectors at the specific stage.

### Intra-Golgi Transport

The Golgi complex is composed of cis-, medial-, trans-cisternal, and the TGN. After entering the Golgi, proteins or other molecules are transported from the cis face to the TGN (intra-Golgi transport), and are then sorted to determine their final destination (Boncompain and Weigel, 2018). Rab19, Rab33, Rab34, and Rab36 are localized to the distinct compartments of Golgi, and may be involved in the anterograde transport of intra-Golgi, while Rab6 is a key determinant of retrograde trafficking in Golgi (Galea and Simpson, 2015). Of note, Rho family GTPase Cdc42 can modulate bi-directional Golgi transport by targeting the dual functions of COPI vesicles, and this effect is controlled by environmental cues (Park et al., 2015). Actually, the mechanisms behind intra-Golgi transport remain poorly understood.

### Golgi-to-Cell Surface Transport

The transportation of TGN to cell surface is directed by exocyst, which is a multimeric complex that associates with the TGN and the plasma membrane. The assembly of the exocyst complex is mainly regulated by RalA and RalB. In addition, Rab (Rabs3, 8, 11, and 27), Rho (Cdc42, Rho1, Rho3, and RhoQ) and Arf (Arf6) family proteins can also interact with exocyst subunits to modulate its function (Wu and Guo, 2015; Nishida-Fukuda, 2019). Then the vesicles are secreted with the coordination of Arf6 (Lawrence and Birnbaum, 2003; Pelletan et al., 2015). Moreover, Rab10, Rab13, Rab14, Rab26, Rab31, and Rab39 in Rab family GTPases (Bhuin and Roy, 2014; Galea and Simpson, 2015), and RhoD in Rho family GTPases (Olayioye et al., 2019), as well as Arl1, Arl8b in Arl family GTPases (Tuli et al., 2013; Yu and Lee, 2017), are also required for the trafficking of TGN to cell surface.

### Golgi-to-Endocytic Compartment Transport

Endocytic compartments (endosomes or lysosomes) are another destination for vesicles from TGN, since the proteins such as MPR and lysosomal associated membrane protein (LAMP) function in late endosomes or lysosomes (Boncompain and Weigel, 2018). The MPR transport from TGN to endocytic pathway is regulated by Arf1 GTPase (Waguri et al., 2003). And Rab31 is another GTPase that thought to play a role in TGN-to-endosome trafficking, as evidenced by the colocalization of Rab31 with TGN and endosomes, also by the involvement of Rab31-containing vesicular organelles in TGN-to-endosome transports (Rodriguez-Gabin et al., 2001).

### Small GTPases in Autophagic Pathway

Autophagy is an evolutionary conserved process that eliminates defunct proteins and organelles to maintain cellular homeostasis. This pathway comprises the processes for autophagosome formation and maturation, as well as protein secretion mediated by autophagy (exophagy), and is achieved by small GTPase-mediated dynamic membrane trafficking and membrane interaction (Soreng et al., 2018).

### Autophagosome Formation Step

The autophagosome is formed by the following ordered steps: initiation of autophagy, formation of the phagophore, expansion, and elongation of the phagophore membrane, ultimate closure of phagophore to become an autophagosome (Soreng et al., 2018). These processes are largely exerted by the autophagy-related (ATG) proteins [reviewed in (Yu et al., 2018)], and are also regulated by multiple small GTPases. For the initiation of autophagy, Rab5 and Arf1 remove mammalian/mechanistic target of rapamycin complex 1 (mTORC1) from lysosomes to the cytosol, thus inhibiting mTORC1 activity and promoting autophagy initiation (Li et al., 2010). Rab12-mediated trafficking promotes the degradation of mTORC1 activator, then stimulates the autophagy induction as well (Matsui and Fukuda, 2013). While other small GTPases including Ras-related GTP-binding protein A and B (RagA and RagB) suppress the autophagy initiation by delivering mTORC1 to a location where it can be activated (Sancak et al., 2008). The phagophore is formed with membranes that from membranous organelles (Nakatogawa, 2020). This process is mediated by Arf1 (Gui et al., 2019), Arf6 (Moreau et al., 2012), Rab1a,1b (Zoppino et al., 2010; Lipatova et al., 2012; Mochizuki et al., 2013), Rab2 (Ding et al., 2019), Rab6 (Yang and Rosenwald, 2016), Rab9 (Nishida et al., 2009; Saito et al., 2019), Rab11 (Longatti et al., 2012; Puri et al., 2018), Rab30 (Nakajima et al., 2019), Rab32 (Hirota and Tanaka, 2009), and Arl1 (Yang and Rosenwald, 2016), while Rab39 functions as a negative regulator of this process (Seto et al., 2013). Then the phagophore membrane expands and elongates with the assistance of Arf1 (van der Vaart et al., 2010), Rab5 (Dou et al., 2013), Rab14 (Mauvezin et al., 2016), Rab26 (Binotti et al., 2015), Rab33b (Itoh et al., 2008), and Rab37 (Sheng et al., 2018). Finally, Rab5 promotes the phagophore closure catalyzed by endosomal sorting complex required for transport (ESCRT) through recruiting ESCRT subunit Snf7 to Atg17-decorated open phagophores (Zhou et al., 2019).

### Autophagosome Maturation Step

The autophagosome maturation process mainly refers to the fusion between autophagosomes with endosomes (early and late endosomes) and lysosomes, while the distribution of lysosomes and the delivery of lysosomal proteins or hydrolases are membrane trafficking events that can also affect the maturation of autophagosomes, and all these processes are controlled by small GTPases. Because the process for autophagosome maturation is quite similar to the endosome maturation in endocytic pathway, thus it is not surprising that small GTPases functioning in endosome maturation also regulate the maturation of autophagosomes. Additionally, the autophagosome-endosome/lysosome fusion step largely relies on Rab7 (Stroupe, 2018), but also requires Rab2 (Ding et al., 2019), Rab9 (Nozawa et al., 2012; Szatmari et al., 2014), Rab11 (Szatmari et al., 2014), Rab14 (Mauvezin et al., 2016), Rab21 (Jean et al., 2015), Rab24 (Amaya et al., 2016), and Rab33b (Itoh et al., 2008). Moreover, the lysosomal transport toward cell periphery is mainly determined by Arl8b (Korolchuk et al., 2011; Adnan et al., 2020). And the delivery of lysosomal membrane protein to lysosomes is dependent on Rab2 (Lund et al., 2018), while Rab6 selectively promotes the delivery of hydrolases, but not other lysosomal proteins, such as V-ATPase subunits or LAMP (Ayala et al., 2018).

### Exophagy Step

Emerging evidence demonstrates that a plethora of factors (mainly the leaderless proteins that lack an ER-signal peptide) are secreted in an autophagy-dependent manner, and this process is exophagy (Abrahamsen and Stenmark, 2010; Cavalli and Cenci, 2020). A study revealed that interferon (IFN)-γ-induced exophagy of annexin A2 (ANXA2) is dependent on Rab8a, Rab11, and Rab27a (Chen et al., 2017). Another study reported that IL-1β secretion caused by autophagy induction is relied on Rab8a (Dupont et al., 2011). Since these GTPases (Rab8a, Rab11, and Rab27a) are also involved in other membrane trafficking process such as exocytic pathway, the findings that these GTPases modulate the exophagic pathway implicate a cooperation between exophagy with other membrane trafficking pathways. In addition, the role of other small GTPases in exophagy and the underlying mechanisms of these GTPases require further investigation.

## Regulation of Small GTPases by Ubiquitination

As mentioned above, small GTPases play an important role in multiple membrane trafficking processes, thus the modulation of these GTPases is the key determinant for exerting their functions. Studies have reported that small GTPase stability, activity, and localization can be regulated by ubiqutination (Table 1).

**TABLE 1:** E3s and DUBs regulating small GTPase stability, activity, or localization.

Small GTPases	Ubiquitination site (s)	E3s	DUBs	References
**E3s and DUBs regulating small GTPase stability**				
RhoA	Lys6, 7	Smurf1		Wang et al., 2003; Tian et al., 2011
GDP-RhoA		Cullin3-BACURD		Chen et al., 2009
Total RhoA, GTP-RhoA		Fbxw7		Li J. et al., 2016
Total RhoA, GTP-RhoA, and GDP-RhoA	Lys135	SCF		Wei et al., 2013
GTP-RhoA			OTUB1	Edelmann et al., 2010
RhoB	Lys6, 7	Smurf1		Wang et al., 2014
RhoB	Lys162, 181	Cullin3-Rbx1		Xu et al., 2015; Kovacevic et al., 2018
Rac1	Lys147	XIAP, cIAP1		Oberoi et al., 2012
GTP-Rac1		HACE1		Torrino et al., 2011; Daugaard et al., 2013
Rac1	Lys166	SCF^*FBXL19*^		Zhao et al., 2013
Rac3	Lys166	SCF^*FBXL19*^		Dong et al., 2014
Cdc42	Lys166	XIAP		Murali et al., 2017
Rab35				Villarroel-Campos et al., 2016
GDP-Rab8				Takahashi et al., 2019
Rab27a				Song et al., 2019
Arl4c and Arf6		Cullin5		Han et al., 2020
**E3s and DUBs regulating small GTPase activity**				
Rab5	Lys140, 165			Shin et al., 2017
Rab7	Lys38	Parkin		Song et al., 2016
Rab7	Lys191		USP32	Sapmaz et al., 2019
Rab11a	Lys145	HACE1-β2AR		Lachance et al., 2014
**E3s and DUBs regulating small GTPase localization**				
Rab7	Lys191		USP32	Sapmaz et al., 2019
Rab7	Lys38	Parkin		Song et al., 2016
RhoB	Lys162, 181	Cullin3-Rbx1		Kovacevic et al., 2018
GTP-RhoA, GTP-Rac1, and GTP-Cdc42			USP17	de la Vega et al., 2011
RalA and RalB				Neyraud et al., 2012

### Ubiquitination Regulates Small GTPase Stability

As two major protein degradation pathways, the ubiquitin-proteasome system (UPS) and autophagy are critical for the maintenance of protein homeostasis in cells, and they recognize ubiquitin as a degradation signal (Pohl and Dikic, 2019). The UPS mainly recognizes K48-linked polyubiquitin chains conjugated on single and short-lived proteins and targets them to proteasome for degradation, while autophagy sequesters K63- and K48-linked polyubiquitin chains associated long-lived proteins to autophagosomes and ultimately fuse with lysosomes for degradation. The modulation of small GTPases by ubiquitination plays an important role in membrane trafficking processes, and the proteolysis of them mostly relies on the UPS.

The most well-studied GTPase that is degraded by the UPS is RhoA. Specifically, RhoA can be polyubiquitin-modified by the SMAD-specific E3 ubiquitin protein ligase 1 (Smurf1) on lysine (Lys) 6 and Lys7 residues, thus resulting in the degradation of RhoA (Wang et al., 2003; Tian et al., 2011). Cullin3-BACURD ubiquitin ligase complex selectively interacts with GDP-bound RhoA, rather than GTP-bound or nucleotide free RhoA, to mediate its ubiquitination and proteasomal degradation (Chen et al., 2009). Different from the Cullin3-BACURD ubiquitin ligase, F-box and WD repeat domain-containing7 (Fbxw7) E3 ubiquitin ligase complex mediates the ubiquitination and degradation of the total RhoA and the active GTP-RhoA (Li H. et al., 2016). Additionally, Skp1-Cullin1-F-box (SCF) E3 ligase catalyzes the ubiquitination of the total, active and inactive forms of RhoA on Lys135 and then promotes the degradation of RhoA, which is dependent on the phosphorylation of RhoA by Erk2 (Wei et al., 2013). On the contrary, the ubiquitination modification of RhoA can be removed by deubiquitinating enzymes (DUBs). For instance, during *Yersinia* infection, otubain 1 (OTUB1) can disassemble the Lys48-linked polyubiquitin chains from GTP-RhoA to maintain its stability (Edelmann et al., 2010).

Additionally, the stability of other Rho GTPases encompassing RhoB, Rac1, Cdc42 and atypical Rho GTPases, is also controlled by the UPS. RhoB is polyubiquitinated for proteasomal degradation mainly by two E3 ligases. Smurf1 promotes RhoB degradation by conjugating ubiquitin on its Lys6 and Lys7 residues (Wang et al., 2014), while Cullin3-Rbx1 E3 ligase complex transfers K63 polyubiquitin chain to Lys162 and Lys181 of RhoB to promote its lysosomal localization and degradation via a proteasomal as well as a lysosomal pathway (Xu et al., 2015; Kovacevic et al., 2018). Moreover, inhibitors of apoptosis proteins (IAPs), including X-linked IAP (XIAP), and cellular IAP1 (c-IAP1), usually target Rac1 to catalyze its polyubiquitination on Lys147 site and then promote its degradation (Oberoi et al., 2012). Meanwhile, HECT domain and Ankyrin repeat containing E3 ubiquitin-protein ligase (HACE1) preferentially binds to the active form of Rac1 (GTP-Rac1) for its degradation (Torrino et al., 2011; Daugaard et al., 2013). The stability of Rac1 and Rac3 are also regulated by SCF^*FBXL*19^ complex that transfers polyubiquitin chains to Lys166 residue of phosphorylated Rac1 or Rac3 to promote their proteasomal degradation (Zhao et al., 2013; Dong et al., 2014). And the Lys166 residue of Cdc42 can also be conjugated with polyubiquitin chains by XIAP, and this modification provide a signal for its proteasomal degradation (Murali et al., 2017).

Among the Rab GTPases, Rab35 is the first protein reported to be degraded by the UPS, which is controlled by p53-related protein kinase (PRPK) and microtubule associated protein 1B (MAP1B) (Villarroel-Campos et al., 2016). Rab8 can also be targeted by the UPS, but only GDP-Rab8a is rapidly degraded with the assistance of BAG6 (BAT3/Scythe), while GTP-Rab8a is highly stable (Takahashi et al., 2019). The accumulated GDP-bound Rab proteins tend to form aggregates in the cytoplasm due to the exposure of hydrophobic groups, so the clearance mechanism of inactive GDP-Rab proteins is critical for maintaining cellular homeostasis. However, the specific E3 ubiquitin ligase of GDP-Rab8a is not identified. As BAG6 can associate with E3s including RNF126 and gp78 (Xu et al., 2013; Rodrigo-Brenni et al., 2014), it is possible Rab8a may be a substrate of BAG6-associated ubiquitin ligase. Another recent study demonstrates that the kidney and brain protein (KIBRA) can interact with Rab27a, and then prevents the ubiquitination-mediated degradation of Rab27a (Song et al., 2019). In addition, the stability of Arf GTPases has also been reported to be regulated by UPS. An example is that the protein levels of Arl4c and Arf6 are downregulated in the presence of Cullin5 E3 ligase (Han et al., 2020). Since UPS can mediate the degradation of specific GTP-bound, GDP-bound or total GTPases, it is reasonable that the activity of GTPases is altered accompanied by the degradation of these proteins.

### Ubiquitination Regulates Small GTPase Activity

The conversion between GDP- and GTP-bound GTPases is the key determinant in exerting their functions. Upon being formed, GTP-bound GTPases can recruit specific effectors to regulate cellular activities. Based on this, ubiquitination may regulate small GTPase activity through two modes, including the exchange of GDP- and GTP-bound GTPases, and the recruitment of effectors. And this ubiquitination-mediated regulatory functions have been demonstrated in several Rab GTPases.

**FIGURE 2:**
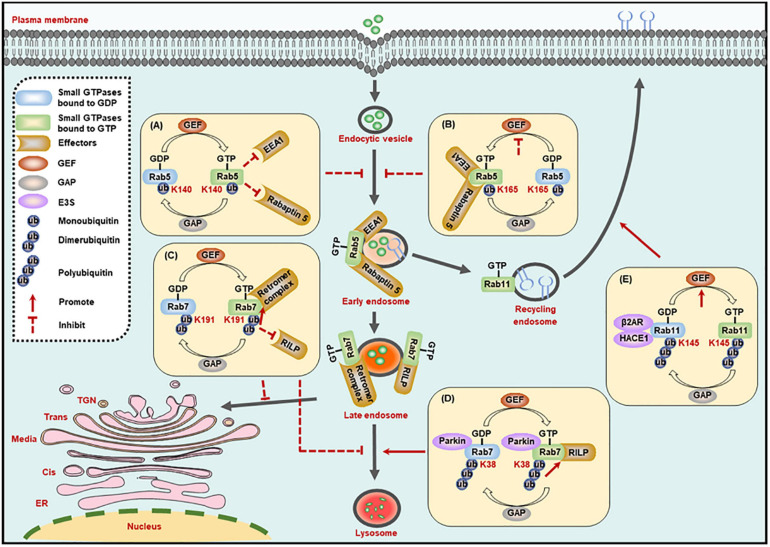
Ubiquitination regulates the activity of small GTPases in the membrane trafficking processes. **(A,B)** The monoubiquitination of Rab5 inhibits its interaction with effectors **(A)**, or its exchange of GDP- to GTP-bound form **(B)**, to suppress the formation of Rab5-positive early endosomes. **(C)** The dimerubiquitination of Rab7 inhibits its interaction with RILP to suppress the motility of late endosomes to lysosomes, while promoting its affinity to retromer complex to suppress the recycling from late endosomes to TGN. **(D)** The polyubiquitination of Rab7 promotes its interaction with RILP to enhance the motility of late endosomes to lysosomes. **(E)** The polyubiquitination of Rab11 promotes its exchange of GDP- to GTP-bound form to enhance the receptor recycling to cell membrane. GEF, guanine nucleotide exchange factor; GAP, GTPase-activating protein; K, Lysine (Lys).

The early endosome marker Rab5, a key member of the Rab family, is crucial in endocytosis and membrane transport. Rab5 could be monoubiquitinated on three residues, including Lys116, Lys140, and Lys165, among which, the monoubiquitination on Lys140 and Lys165, but not Lys116, suppresses the activity of Rab5. Specifically, monoubiquitination on Lys140 impairs the interaction between Rab5 and its downstream effectors such as Rabaptin 5 and early endosome antigen 1 (EEA1), and Lys165 monoubiquitylation hinders GEF-mediated guanine nucleotide conversion, thereby leading to the blockade of endocytic pathway (Figures 2A,B; Shin et al., 2017). It is worth mentioning that the ubiquitination site is critical to determine small GTPase activity.

Rab7 activity is also regulated by its ubiquitination. The dimerubiquitination on the Lys191 residue of Rab7 blocks its interaction with RILP, thus inhibiting Rab7-mediated late endosome motility and the perinuclear localization of the late compartment. In turn, the DUB enzyme ubiquitin-specific protease 32 (USP32) removes the ubiquitin chains from Lys191, and then restores the late endosome motility and the perinuclear localization of the late compartment mediated by Rab7 (Sapmaz et al., 2019). Most importantly, the ubiquitination of Rab7 on Lys191 residue can also inhibit the recycling from late endosomes to TGN by enhancing the interplay between Rab7 and the retromer complex and thus stabilize the retromer complex on endosomes (Figure 2C; Sapmaz et al., 2019). In addition, polyubiquitinated Rab7 on the Lys38 residue by the E3 ligase Parkin exhibits stronger affinity for its effector RILP, and the high affinity improves the activity and membrane localization of Rab7 (Figure 2D; Song et al., 2016).

Another example regarding ubiquitination-dependent regulation of small GTPase activity is Rab11a, which mediates the receptor recycling, and its activity is regulated by ubiquitination catalyzed by HACE1 and a scaffold β2AR. The HACE1-β2AR complex-catalyzed Rab11a ubiquitination on Lys145 destroys the interaction between β2AR and GDP-bound Rab11a, and then releases Rab11a to combine GTP and thus promotes its activation (Figure 2E). But the mechanism that ubiquitination of Rab11a leads to its activation needs further determination. In addition, co-expression of HACE1 and β2AR also potentiates the ubiquitination of Rab6a and Rab8a. Considering the functional connection of Rab6a, Rab8a, and Rab11a in intracellular transport, whether the ubiquitination of these three proteins will affect each other remains to be defined (Lachance et al., 2014).

### Ubiquitination Regulates Small GTPase Localization

Small GTPases are usually localized on the cytosol in their inactive GDP-bound form. Accompanied with the conversion between GDP- and GTP-bound forms, GTPases are transferred from the cytosol to specific membranes to recruit effectors for functioning (Stenmark, 2009). Thus, the ubiquitination-dependent regulation of small GTPase activity may also plays important roles in modulating their localizations. For example, ubiquitination of Rab7 on Lys191 residue blocks late endosome motility and the perinuclear localization of the late compartment, and this modification also facilitates the membrane localization of Rab7, as evidenced by the increased membrane-to-cytosol ratio (Sapmaz et al., 2019). Another study indicates that Parkin catalyzes the ubiquitination of Rab7 to enhance its binding affinity for RILP, and thus ultimately promoting the membrane localization of Rab7 (Song et al., 2016).

Moreover, the localization of Rho and Ras family GTPases is also modulated by ubiquitination. For instance, the ubiquitination of RhoB on Lys162 and Lys181 can direct RhoB to lysosome for degradation (Kovacevic et al., 2018). And the ubiquitin-specific protease 17 (USP17) is required for the membrane localization of active Rho GTPases, including RhoA, Rac1, and Cdc42, while the underlying molecular mechanism is still unclear (de la Vega et al., 2011). Furthermore, the ubiquitination of Ras GTPases (including RalA and RalB) can provide a signal for their localization. Specifically, ubiquitination directs RalA transport to plasma membrane, while deubiquitination of RalA occurs in lipid raft microdomains and promotes raft endocytosis (Neyraud et al., 2012). Up to now, the role and the underlying molecular mechanism by which ubiquitylation regulate the location of the small GTPases remain obscure and warrant further investigation.

## Dysregulation of Small GTPase Ubiquitination in Membrane Trafficking-Related Diseases

**FIGURE 3:**
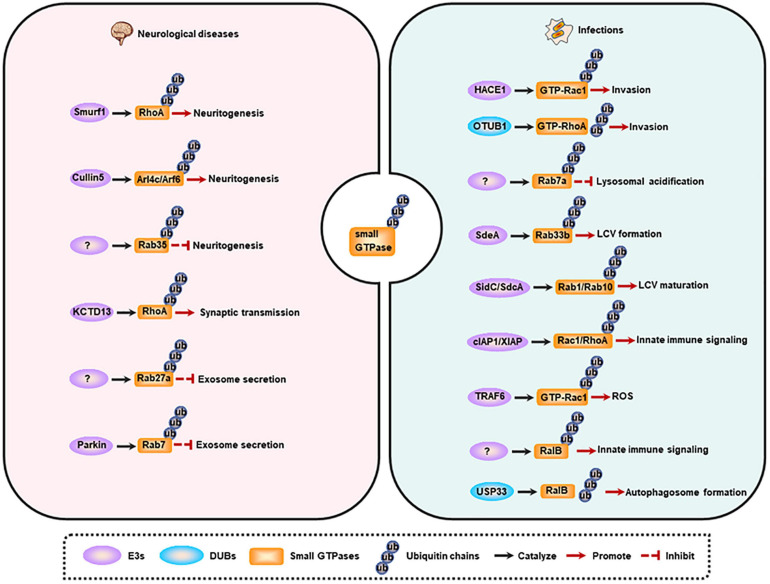
Small GTPase ubiquitination and membrane trafficking-related diseases. Left, small GTPase ubiquitination is involved in neuritogenesis, synaptic transmission, and exosome secretion. Dysregulation of these processes contributes to the occurrence of neurological diseases. Right, small GTPase ubiquitination regulates pathogen invasion, lysosomal acidification, LCV maturation, innate immune signaling activation, ROS production, and autophagy induction. Dysregulation of these processes contributes to the occurrence of infections. LCV, *Legionella-*containing vacuole; ROS, reactive oxygen species.

Membrane trafficking is the key determinant for cellular activities, including neurodevelopment and host immune response against pathogens. The trafficking is a complex, dynamic, but an ordered process, which is mainly controlled by small GTPases’ spatiotemporal alterations that potentially dependent on ubiquitination (as described above). Consequently, the dysregulation of small GTPase ubiquitination will cause multiple human membrane trafficking-related diseases, and here we focus on the neurological and infectious diseases (Figure 3).

### Dysregulation of Small GTPase Ubiquitination in Neurological Diseases

The nervous system is the commander of multitude biogenic activities, and neurodevelopment is critical for this ability. The entire neurodevelopmental process, including neurogenesis, neuritogenesis, synaptogenesis, differentiation, synaptic plasticity, synaptic transmission, and aggregate secretion, depends on the membrane trafficking, and is regulated by small GTPases [reviewed in (Qu et al., 2019)]. Emerging evidence shows that small GTPase ubiquitination is a more precise mechanism for modulating neurodevelopment at the specific stage. Moreover, the alteration of this protein modification accounts for many neurological disorders.

Neuritogenesis has been reported to be regulated by small GTPase ubiquitination. Neuritogenesis is inhibited by RhoA, and Smurf1 can interact with RhoA and mediate its ubiquitination and degradation, thus enhancing neurite outgrowth (Bryan et al., 2005; Deglincerti et al., 2015). Some other small GTPases including Arl4c and its effector Arf6 are also negative regulators of neuritogenesis (particularly dendritogenesis), and are ubiquitinated and degraded by Cullin-5 under physiological conditions. Furthermore, depletion of Cullin-5 causes the formation of V-shaped dendrites that usually appears in neurodegenerative models or in the brains of Alzheimer’s disease (AD) patients (Han et al., 2020). However, Rab35 favors axon elongation in rat primary neurons, and this process is controlled by the proteolysis of Rab35 in a UPS-dependent manner (Villarroel-Campos et al., 2016). Additionally, synaptic transmission is also modulated by small GTPase ubiquitination. RhoA can prevent synaptic transmission, and deletion of its E3 ligase KCTD13 results in accumulated RhoA, which in turn reduces functional synapse number and synaptic transmission. Consistently, treatment with RhoA inhibitor rhosin can reverse the reduced synaptic transmission in *Kctd13*-deleted mice, implicating that RhoA is a potential therapeutic target for neuropsychiatric disorders (Escamilla et al., 2017).

The abnormal aggregation of misfolded proteins is the pathological character of neurodegenerative diseases, and these proteins can be delivered to non-effected regions by exosomes, thus promoting the progression of the disease (Jan et al., 2017). The secretion of exosomes in neural cells is another process that is controlled by small GTPase ubiquitination. Evidence indicates that KIBRA and Rab27a are predominantly expressed in the brain, and KIBRA-mediated inhibition of Rab27a ubiquitination and degradation promotes the secretion of exosomes, suggesting that decreased Rab27a ubiquitination may be involved in the initiation and progression of neurodegenerative diseases (Song et al., 2019). Another study shows that Parkin mediates the ubiquitination of Rab7, and then promotes its affinity for RILP, eventually directing Rab7 to the retromer pathway. While the deregulation of Rab7 ubiquitination in Parkin-deficient cells results in the impairment of the retromer function, but leading to the increased secretion of exosomes, a phenomenon observed in Parkinson disease (PD). These findings raise a possibility that increasing Rab7 ubiquitination may be a potential therapeutic strategy for PD (Song et al., 2016).

### Dysregulation of Small GTPase Ubiquitination in Infections

Membrane trafficking is vital for host immune response against pathogens. Based on this, trafficking events, such as phagocytosis and the following phagosome maturation, together with autophagy, can be prevented, or hijacked by pathogens (especially intracellular pathogens) to benefit their survival (Inoue et al., 2018; Chai et al., 2020). Moreover, increasing studies have demonstrated that the ubiquitin system is another sensitive target of many bacterial pathogens (Li J. et al., 2016). As small GTPases are the main modulators in membrane trafficking, the ubiquitination of these proteins has also been reported to be manipulated by pathogenic microorganisms.

The invasion into host cells is fundamental for intracellular pathogens to establish an infection, and Rho family GTPases are crucial for this process (Visvikis et al., 2011). Targeting Rho GTPases to regulate their ubiquitination is the strategy of pathogens for entering the host cells. Specifically, effectors or toxins produced by pathogenic microorganisms interfere with the ubiquitination of Rho GTPases. For instance, cytotoxic necrotizing factor-1 (CNF1) from *Escherichia coli* can induce the activation of Rac1, and then promotes the HACE1-mediated ubiquitination and degradation of GTP-bound Rac1, leading to an increased invasion of pathogens toward endothelial cell monolayer (Torrino et al., 2011). While prior to *Yersinia* infection, wild-type OTUB1 catalyzes the deubiquitination of active-form RhoA to stabilize the protein, and then enhances the cellular susceptibility to invasion. However, once virulence factor *Yersinia* serine/threonine kinase A (YpkA) is secreted into the infected cells, it can interact with phosphorylated OTUB1 as well as GDP-bound RhoA to block active RhoA formation, and ultimately preventing further bacterial uptake. And the limiting invasion efficiency of bacteria may contribute to a decreased intracellular killing mediated by host immune defense (Edelmann et al., 2010). Additionally, a ubiquitinome study of *Salmonella*-infected epithelial cells has revealed that the ubiquitination level of Cdc42 and RhoG is upregulated, whereas Rac1 ubiquitination is decreased, at early-stage of infection. These effects may benefit the invasion of the pathogen. However, the exact functional output of these modifications and the E3s as well as DUBs of these proteins need further research in the future (Fiskin et al., 2016).

To replicate in the cell, pathogenic microorganisms can manipulate the host’s membrane trafficking pathways to avoid degradation and to shape a replication-permissive niche, such as *Salmonella*-containing vacuoles (SCV) for *Salmonella enterica*, and *Legionella-*containing vacuole (LCV) for *Legionella pneumophila* (*L. pneumophila*). Upon *Salmonella* infection, Rab7a is highly ubiquitinated by an unknown E3 ligase, which may contribute to the inhibition of lysosomal acidification and the subsequent degradation (Fiskin et al., 2016). Furthermore, effectors released by *L. pneumophila* have evolved ubiquitinating enzyme activities to mediate the ubiquitination of host proteins directly. During infection, effector protein SdeA secreted by *L. pneumophila* can promote the ubiquitination of ER-associated protein Rab33b, leading to an increased intracellular bacterial replication by facilitating LCV formation (Qiu et al., 2016). Rab10 can be recruited to the LCV and then be ubiquitinated by SidC/SdcA to promote LCV maturation (Jeng et al., 2019). Rab1, a GTPase that is localized on the LCV, can also be ubiquitinated in a SidC/SdcA-dependent manner and then play key roles in LCV maturation (Horenkamp et al., 2014).

Other host immune responses related to small GTPase ubiquitination can also be manipulated by pathogens. For example, CNF1-induced ubiquitination-mediated proteasomal degradation of activated Rho proteins limits the production of inflammatory proteins and immunomodulators, thus attenuating host cell immune responses (Munro et al., 2004). Another example is that VopS from *Vibrio parahaemolyticus* hinders the interaction of Rho GTPases (Rac1 and RhoA) with their E3 ligases (cIAP1 and XIAP), may causing a suppression to host immune response such as collapse of the actin cytoskeleton, inactivation of NF-κB, Erk and JNK pathways (Woolery et al., 2014). Additionally, in response to pathogen infection, E3 ubiquitin ligase TRAF6 selectively interacts with inactive Rac1T17N. Once the GDP-dissociation inhibitor (GDI) is dissociated from Rac1, this will promote the charging of GTP on Rac1. Under this condition, TRAF6 can further catalyze K63-linked polyubiquitination of GTP-Rac1, eventually inducing the recruitment of mitochondria to phagosome and the subsequently formation of ROS in macrophages for pathogen killing (Geng et al., 2015). And RalB ubiquitination is critical for its binding to exocyst complex component 2 (EXOC2) and the triggered innate immune signaling, while deubiquitination of RalB mediated by ubiquitin-specific protease 33 (USP33) facilitates the assembly of the RalB-Exocyst complex component 84 (EXO84)-beclin-1complex to drive autophagosome formation (Simicek et al., 2013). These studies suggest that the ubiquitination of small GTPases is involved in multiple steps of pathogen infections.

## Conclusion

Membrane trafficking is tightly regulated to maintain cellular homeostasis, and defects in the regulatory machinery of this process leads to human diseases. This review discusses the modulation of membrane trafficking pathways by small GTPases, and the regulation of small GTPases by ubiquitination as well as the associated human diseases (focusing on the neurological diseases and infections). Three key points are extracted from this review: first, there is a crosstalk between different regulatory pathways, which may lead to synergistic or antagonistic effects; second, the regulatory function of ubiquitination on small GTPases is not singular due to the interactions among GTPase stability, activity and localization; third, the ubiquitination levels of small GTPases may be altered during the progression of diseases, especially the infections, as pathogens can manipulate host immune response to benefit their intracellular survival through multiple strategies. Moreover, emerging ubiquitinome studies have revealed that ubiquitination of small GTPases is a prominent characteristic of multiple human diseases (Fiskin et al., 2016; Jeng et al., 2019). Additionally, the ubiquitination site is critical to decide small GTPase activities (Shin et al., 2017). Therefore, identifying the ubiquitination sites and their output functions during the progression of diseases will provide new insights into novel therapeutics for these diseases. Furthermore, several other important issues also warrant further study to provide clearer and more comprehensive picture for disease treatment. For instance, what are the E3s and DUBs of small GTPases? If E3 or DUBs are effectors from pathogens, whether they regulate host protein substrates and their functions by novel mechanisms different from their host counterparts? Moreover, various types of atypical ubiquitination have been revealed, and what are their regulatory roles on small GTPases and membrane trafficking, and whether there is a crosstalk between different ubiquitination modifications? Thus, there are many exciting questions waiting to be clarified, and a more in-depth understanding of the molecular mechanisms by which ubiquitination regulates small GTPases and the membrane trafficking process can provide new insights and novel targets for the development of more effective and specific therapeutics for membrane trafficking-related human diseases.

## Author Contributions

All authors listed have made a substantial, direct and intellectual contribution to the work, and approved it for publication.

## Conflict of Interest

The authors declare that the research was conducted in the absence of any commercial or financial relationships that could be construed as a potential conflict of interest.
